# Chemosensory dysfunction in Cushing’s syndrome

**DOI:** 10.1007/s12020-021-02707-z

**Published:** 2021-04-05

**Authors:** Elena Heger, German Rubinstein, Leah T. Braun, Stephanie Zopp, Jürgen Honegger, Max Seidensticker, Martin Reincke, Andrea Oßwald

**Affiliations:** 1 Medizinische Klinik und Poliklinik IV, Ludwig-Maximilians-Universität, München, Germany; 2 Department of Neurosurgery, University of Tuebingen, Tübingen, Germany; 3 Klinik und Poliklinik für Radiologie, Ludwig-Maximilians-Universität, München, Germany

**Keywords:** Cushing’s disease, Cortisol, ACTH, “Sniffin’ Sticks”, “Taste Strips”

## Abstract

**Purpose:**

Cushing’s syndrome (CS) can lead to structural changes in the brain and cognitive impairment, but chemosensory function has not been investigated yet. The aim was to analyze sense of smell and taste in patients with CS and explore the effect of therapy.

**Methods:**

The study cohort comprised 20 patients with florid CS treated between 2018 and 2020 in the outpatient clinic of the LMU Munich. We compared these 20 patients with CS to 40 healthy subjects matched for age, sex, and smoking status. Patients’ sense of smell and taste was examined at diagnosis and 3 months after successful therapeutic surgery leading to clinical and biochemical remission. Odor threshold, discrimination, and identification were measured with “Sniffin’ Sticks”, taste was measured with “Taste Strips”. Perceived sense of smell and taste was retrieved via a questionnaire.

**Results:**

Patients with florid CS had significantly reduced smell (total smell score 30.3 vs. 34.4, *p* < 0.0005) and taste scores (9.5 vs. 12.0, *p* < 0.0005) compared to controls and significantly more frequently hyposmia (55 vs. 2.5%, *p* < 0.0005), hypogeusia (40 vs. 0%, *p* < 0.0005), and self-reported chemosensory impairment (60 vs. 0%, *p* < 0.0005). Three months after successful surgery, CS patients showed significant improvement of odor threshold (8.1 vs. 7.0, *p* < 0.0005), odor discrimination (12.0 vs. 11.0, *p* = 0.003), total smell score (33.4 vs. 30.3, *p* < 0.0005), and taste (11.5 vs. 9.5, *p* = 0.003).

**Conclusions:**

Chemosensory dysfunction is a novel and clinically relevant feature of CS.

## Introduction

Excessive cortisol production causes endogenous Cushing’s syndrome (CS). It is a severe disease with increased cardiovascular mortality and a multitude of possible symptoms [1]: patients mainly suffer from cardiometabolic changes like hyperglycemia, hypertension, and central obesity. But mental health issues and a permanent change of brain morphology and cognitive function can occur as well [2, 3]. Consequently, patients report a decreased quality of life [4].

First-line therapy for CS is the removal of the hormone producing tumor [5]. This requires careful diagnosis and subtyping, as tumor location and produced hormones vary between CS subtypes: the ACTH-dependent CS is further subdivided into the ACTH-secreting pituitary adenoma (Cushing’s disease (CD)) which is the most common form of CS and the non-pituitary ectopic form (ECS). The main subtype for ACTH-independent CS are unilateral cortisol producing adrenal adenomas (CPA) that autonomously produce cortisol [6].

The changes in brain structure and impairment of cognitive function observed in CS [7] motivated us to explore changes of smell and taste similar to neurodegenerative diseases, in which a loss of the sense of smell and taste is associated with disease progression and can even lead to early diagnosis [8, 9]. Additionally, treatment with exogenous glucocorticoids like dexamethasone is known to lead to diminished sense of smell and taste [10, 11]. However, this sensory function has not been measured in patients with CS so far.

Therefore, our goal was to analyze sense of smell and taste in CS patients and examine the effect of therapy.

## Materials and methods

### Patient cohort

From 2018 to 2020, we recruited 20 patients with biochemically confirmed endogenous CS (Table 1), treated in the German Cushing’s Registry CUSTODES (Cushing Syndrom: Therapie und Outcome in Deutschland) at the Medizinische Klinik und Poliklinik IV in Munich. All patients had newly diagnosed, florid CS. They were tested twice: initially after being diagnosed before any treatment, and a second time 3 month after curative surgery while being in clinical and biochemical remission. All patients with CD underwent endonasal-transsphenoidal surgery, all patients with CPA had unilateral adrenalectomy and the ECS patient had an occult source of cortisol production and underwent bilateral adrenalectomy. Curative surgery was defined by postoperative adrenal insufficiency requiring glucocorticoid replacement therapy with hydrocortisone in physiological dose (20–25 mg per day). Patients received standard glucocorticoid replacement therapy postoperatively, and on the day of testing the patients took their usual morning glucocorticoid dose. Exclusion criteria were age (<18 and >70 years), nose and neck surgery in the last 6 months prior to diagnosis, drug abuse, and odor disorders different from CS. The responsible ethics committee of the “Medizinische Fakultät der Ludwig-Maximilians-Universität München” approved the study and all patients confirmed written informed consent. Additionally, we recruited a control group of 40 healthy individuals with the same exclusion criteria, matched to the patients by age-group (18–35, 36–55, >55 years), sex, and smoker/non-smoker classification.

**Table 1:** Clinical characteristics of CS patients at diagnosis

	CS patients before therapy											
Standard values								1.8–24 µg/dl	4–50 pg/ml	<83 µg/24 h	<1.8 µg/dl	<1.5 ng/ml
Age	Sex	Smoker	CS subtype	HbA1c (%)	Weight (kg)	BMI (kg/m^2^)	Blood pressure (mmHg)	Basal level of cortisol	ACTH	UFC	LDDST	LNSC
19	Male	No	CD	5.5	62	22.0	124/70	20.3	109	483	15.1	4.0
58	Female	No	CD	6.1	79	28.0	156/81	20.8	107	203	7.8	5.4
21	Female	No	CD	5.1	65	25.1	116/73	43.7	93	606	5.1	45.4
45	Female	No	CD	5.8	79	27.3	139/91	11.4	87	172	6.3	6.9
59	Female	No	CD	6.0	63	28.0	166/91	21.6	77	164	18.1	4.2
51	Female	Yes	CD	5.2	94	32.9	133/83	15.0	68	782	4.0	3.2
56	Male	No	CD	5.5	72	22.0	176/111	20.6	64	270	5.6	5.7
34	Female	No	CD	5.3	91	31.5	114/81	27.9	63	101	3.0	1.4
61	Female	No	CD	9.4	111	40.8	140/73	20.5	57	148	3.2	2.6
33	Female	Yes	CD	–	62	20.2	119/79	25.6	54	1240	52.8	5.8
44	Female	No	CD	–	117	44.6	132/86	16.0	43	297	18.4	2.6
22	Female	No	CD	–	65	22.2	118/77	26.1	39	372	22.6	3.3
18	Female	No	CD	5.1	89	31.5	114/76	10.9	24	185	11.0	9.3
57	Female	Yes	CPA	5.7	65	29.7	121/79	11.0	8	104	8.0	4.1
59	Female	No	CPA	4.7	83	27.7	127/74	9.1	4	176	11.2	6.9
53	Female	No	CPA	5.5	53	19.5	121/87	7.3	2	167	8.2	3.4
51	Female	Yes	CPA	–	84	30.9	166/96	10.4	2	162	10.5	9.2
37	Female	No	CPA	5.4	64	20.7	136/84	24.1	2	1364	23.3	11.9
18	Female	No	CPA	5.0	67	25.2	148/106	24.5	2	673	14.5	11.5
45	Female	No	ECS	5.9	62	22.8	185/123	32.5	104	789	36.4	6.7

*UFC* 24 h urinary free cortisol, *LDDST* cortisol after low-dose (1 mg) dexamethasone suppression test, *LNSC* Late-night salivary cortisol

### Study protocol

Smell and taste were measured with “Sniffin’ Sticks” [12, 13], respectively “Taste Strips” [14]. Both tests are validated in comparison to previous test sets, show good reproducibility and are internationally established [12, 14–22]. Additionally, we used a questionnaire to assess perceived impairment of smell and taste. The subjects of the control group underwent testing in a similar fashion. For internal validation, we compared the measurements of the “Sniffin’ Sticks” and “Taste Strips” of the control group with data published by Hummel et al. [23] and Landis et al. [16]. Thirty minutes before the measurements, consummation of food and any drinks except water as well as smoking was not allowed. No perfume was allowed on the day of measurement. Measurements were always conducted in the same odorless room.

### Odor testing with “Sniffin’ Sticks”

“Sniffin’ Sticks” are felt tip pens which are filled with odors instead of paint, which are presented 2 cm in front of the patient’s nose with removed cap. The test set examines scores for odor threshold (THR), odor discrimination (DIS), and odor identification (ID). A total smell score (TDI) is evaluated as the sum of THR, DIS, and ID. The examiner wore odorless cotton gloves during testing. For THR and DIS evaluation, the patients were blinded with a sleeping mask as the correct stick had a different color.

The threshold measurement was performed with n-butanol and patients were presented with triplets of pens. The patient was required to identify the only odor filled pen in each triplet, which had a different concentration of n-butanol in each triplet [24]. Triplets were arranged according to a single staircase method and the triplet with the lowest concentration was presented first [18]. The THR score can range from 1 to 16 with steps of 0.25.

In the discrimination test, the patients were presented 16 pen triplets. Each triplet contained two identical odors and one different odor which was supposed to be identified by the patient. The DIS score is the sum of correctly identified pens and can range from 0 to 16 with steps of 1.

In the identification test, the patients were presented with 16 single pens. For each pen the patient had to identify the contained odor from a choice of four common odors. The ID score is the sum of correctly identified odors and can range from 0 to 16 with steps of 1.

The TDI score as sum of THR, DIS, and ID can range from 1 to 48 in steps of 0.25.

For the classification of functional hyposmia [17], we used the updated definition of Hummel et al. [23] where patients with TDI < 30.5 are classified as being hyposmic.

### Taste testing with “Taste Strips”

“Taste Strips” are paper strips that taste sweet, sour, salty, or bitter. The patients tasted 16 “Taste Strips” that contained the four tastes in different concentrations [16]. Patients evaluated each strip with closed mouth and had to make a choice for one of the specific tastes. We used the forced-choice paradigm introduced by Landis et al. [16], which improves the original testing procedure from Mueller et al. [14]. The taste score is the sum of correctly identified tastes and can range from 0 to 16 with steps of 1. The patients were allowed to drink water between different strips. Patients were classified as hypogeusic, if the taste score was below 9 points [14].

### Questionnaire

The questionnaire is provided by the Interdisciplinary Center “Smell & Taste” of the University of Dresden and is recommended by the project group “Olfactology and Gustology” of the German ENT society. Questions examine drug use, chronic illness, exposure to harmful substances, head and neck surgeries, and other reasons for decreased smell or taste. Finally, the questionnaire asked for perceived reduction of both smell and taste (impaired or not impaired).

### Statistical analysis

Test scores of “Sniffin’ Sticks” and “Taste Strips” were compared with Mann–Whitney *U* test (patients before/after treatment vs. control group) and Wilcoxon signed-rank test (patients before vs. after treatment). Results are expressed as median and quartiles. Fisher’s exact test was used to compare binary values (hyposmia, hypogeusia, perceived impairment) between patients and healthy controls and to examine correlations between perceived impairment and hyposmia as well as hypogeusia. Effect size of correlations was reported with Cramer’s *V*. A *p* < 0.05 was considered statistically significant. We calculated that we would need 15 patients and 30 controls to have a power of 0.8 at a *p* level of 0.05. Assuming a dropout rate of 25% during the study we included 20 patients and 40 control subjects. Statistical analysis was performed with standard statistical software (SPSS 21).

## Results

We recruited 20 patients with CS (18 women, 2 men). Thirteen patients had a diagnosis of CD, six patients of CPA, and one patient had the ECS. The CS patients had a median age of 45 years (25th–75th percentile: 28–57 years), 20% were smokers. Table 1 shows the clinical characteristics of the patients. The control group of 40 probands (36 women, 4 men) was matched according to age, sex, and smoking status.

### Reduction of smell and taste in patients with CS

Patients with florid CS had drastically reduced scores of smell (THR, DIS, ID, and total score TDI) and taste compared to the healthy control group (Table 2).

**Table 2:** Smell and taste scores of newly diagnosed florid CS patients, cured CS patients 3 months after surgery and the control group

	CS patients at diagnosis median (25th–75th percentile)	CS patients 3 month after surgery median (25th–75th percentile)	Control group median (25th–75th percentile)	*p* florid CS patients vs. control group	*p* CS patients before vs. after surgery	*p* cured CS patients vs. control group
Smell						
TSH	7.0 (6.3–7.9)	8.1 (7.3–9.0)	8.3 (7.8–8.8)	0.000	0.000	0.403
DIS	11.0 (8.5–12.0)	12.0 (11.0–14.0)	13.0 (12.0–13.0)	0.000	0.003	0.171
ID	12.0 (11.5–14.0)	13.0 (12.0–14.0)	14.0 (13.0–15.0)	0.003	0.148	0.010
TDI	30.3 (25.3–32.9)	33.4 (30.8–35.0)	34.4 (33.6–36.6)	0.000	0.000	0.025
Taste						
Taste score	9.5 (7.0–11.0)	11.5 (10.0–13.0)	12.0 (11.0–13.0)	0.000	0.003	0.103

Mann–Whitney *U* test was used to compare patients to the control group, while the comparison of CS patients before and after treatment was performed with the Wilcoxon signed-rank test

*TSH* odor threshold, *DIS* odor discrimination, *ID* odor identification, *TDI* total smell score

Classification of absolute hyposmia and hypogeusia showed that out of the 20 CS patients, 11 were hyposmic and 8 were hypogeusic (Table 3), compared to 1 and 0 in 40 controls (*p* < 0.0005). Similarly, 12 of 20 patients with CS reported a subjectively perceived reduction of smell and taste at diagnosis, compared to none of the controls (*p* < 0.0005). Every patient with self-reported reduction of smell also reported a reduction of taste and vice versa.

**Table 3:** Subjective and objective impairment of smell and taste in newly diagnosed florid CS patients, cured CS patients 3 months after surgery and the control group

	CS patients at diagnosis	CS patients after surgery	Control group	*p* florid CS patients vs. control group	*p* cured CS patients vs. control group
Subjective					
Perceived impairment of smell and taste	12/20 (60%)	8/20 (40%)	0/40 (0%)	0.000	0.000
Objective					
Hyposmia	11/20 (55%)	5/20 (25%)	1/40 (2.5%)	0.000	0.013
Hypogeusia	8/20 (40%)	2/20 (10%)	0/40 (0%)	0.000	0.107

Comparisons were performed with Fisher’s exact test

### Improvement of sense of smell and taste 3 month following successful surgery in CS patients

**Fig. 1:**
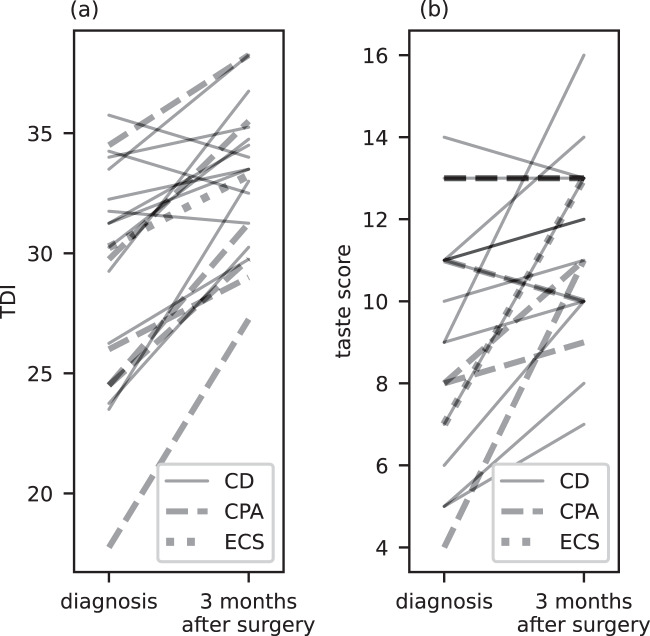
Effect of surgery on smell and taste of individual CS patients. TDI total smell score, CD Cushing’s disease, CPA cortisol producing adenoma, ECS ectopic Cushing’s syndrome. Effect of surgery on smell (**a**) and taste (**b**) of individual CS patients. TDI total smell score, CD Cushing’s disease, CPA cortisol producing adenoma, ECS ectopic Cushing’ssyndrome

Three months after surgery we observed a significant improvement of the taste score as well as the smell scores THR, DIS, and TDI. Improvement of smell identification (ID), however, did not reach statistical significance (Table 2 and Fig. 1). The taste score and the smell scores THR and DIS equaled those of the control group, whereas smell identification and TDI remained reduced (Table 2).

Five of the 20 patients in remission were still hyposmic compared to a single elderly hyposmic proband in the control group (*p* = 0.013, Table 3). Two patients were still classified as hypogeusic. In addition, 8 of the 20 patients in remission still perceived a subjective reduction of smell and taste (*p* < 0.0005 vs. controls).

### Relation between self-reported and objective decrease of smell and taste

**Fig. 2:**
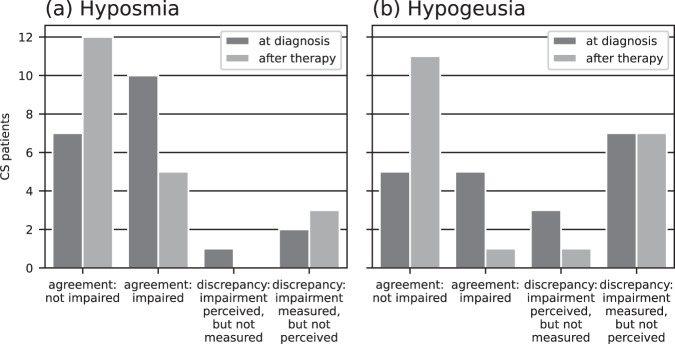
Correlations between objective and subjective impairment of smell and taste in CS patients. Correlations between objective and subjective impairment of smell (**a**) and taste (**b**) in CS patients

All patients either reported an impairment in both smell and taste, or reported no impairment (Fig. 2). While being floridly cushingoid, 10 of 11 hyposmic patients also reported a perceived impairment of smell and taste, while 7 of 9 normosmic patients reported no impairment. Three months after successful surgery, 5 of 5 hyposmic patients were still subjectively impaired, while 12 of 15 normosmic patients did not report any impairment. These correlations between perceived impairment of smell and taste and the classification of hyposmia were significant with a large effect size (Cramer’s *V* > 0.5) for both newly diagnosed patients (*p* = 0.005, *V* = 0.698) and those following successful surgery (*p* = 0.004, *V* = 0.707).

Five of eight hypogeusic floridly cushingoid patients reported a perceived impairment of smell and taste, but also seven patients with normogeusia reported impairment. After therapy, two patients remained hypogeusic, and one reported also a perceived impairment. Of the 18 normogeusic patients, 7 still perceived an impairment of smell and taste. Fisher’s exact test did not detect any correlation between perceived impairment of taste and hypogeusia (*p* = 1.000, *V* = 0.042 for florid patients; *p* = 1.000, *V* = 0.068 after surgery).

## Discussion

We demonstrate that patients with florid CS suffer from relevant impairment of smell and taste. Chemosensory dysfunction is a novel phenotype of CS, which to the best of our knowledge has not been described so far. Since it was a rare finding in our matched control cohort, we believe that self-reported decreased sense of smell and taste could be used as an adjunct diagnostic symptom of CS.

Successful surgical treatment partly restored perceived and objective hyposmia and hypogeusia within 3 months. The reversibility argues for a direct effect of glucocorticoid excess on smell and taste sensing and against a structural effect on the central nervous system.

A reduction of the sense of smell and taste was in our study a frequent problem classifying 55% of patients with florid CS as hyposmic and 40% as hypogeusic. Compared to rheumatoid arthritis, where hyposmia has been reported in 40% of considerably older patients [25], CS seems to impair chemosensory function more strongly. Additionally, 60% of the patients reported a perceived impairment of both smell and taste. Three months after successful surgery, patients could again identify small fragrance concentrations and were able to discriminate scents without significant difference to the control group. The identification of different smells, however, remained impaired and diminished the total smell score. Supposedly, the diminished smell identification is mainly responsible for the remaining hyposmia in 25% of the cured patients.

Although the significantly diminished taste score in florid CS patients improved considerably with therapy, 40% of patients still reported perceived impairment of taste. Notably, patients either reported no impairment or an impairment of both smell and taste. We found a strong correlation between hyposmia and subjective impairment of smell as well as subjective impairment of taste: before and after therapy 85% of the patients’ assessments of impairment matched the hyposmia classification. However, no correlation could be found between hypogeusia and self-reported impairment of taste. Considering that subjective impairment of taste strongly correlates with hyposmia, we conclude that patient’s subjective impairment of taste is based on objective impairment of smell. This supports results by Deems et al. [26], who reported that perceived impairment of taste can usually be attributed to measured impairment of smell in the general population. Consequently, any improvement of the sense of smell (e.g., odor training [27]) should improve perceived smell and taste in CS patients.

Peculiarly, ID as well as perceived sense of smell remained diminished after therapy, while smell discrimination and threshold recovered completely. This implies that the reduced ID score is mainly responsible for the remaining perceived impairment of smell. It is also possible that the ID score generally contributes the most to perceived impairment of smell. This seems plausible, as a missing identification of a common odor can stand out.

This reduced sense of smell and taste in patients with CS has not been reported previously and probably contributes to the reduced quality of life reported in CS patients [28–31].

As a correlation between impairment of smell and depression has been reported [32], possible causes of ID decrease and potential therapies need to be discussed.

Supraphysiologic doses of hydrocortisone and dexamethasone are known to alter the senses of smell and taste [10, 11]. Consequently, it seems likely that the cortisol excess in CS is responsible for the impairment of smell and taste. In addition to the direct influence of cortisol, long-term structural changes in the brains of patients with CS [7] could be responsible for persisting reduction of smell and taste, as an influence of glucocorticoids on, e.g., hippocampus and basal ganglia has been reported [3] with, e.g., a reduced volume of the hippocampus [2].

The impairment of smell identification might be due to a permanent damage or a reversible damage with slow remission. The ID score is the only measured score which requires access to memorized scents. Consequently, if the hippocampus, which is responsible for memory function, is impaired due to the cortisol excess [33, 34], a reduction of the identification of smell could be explained. Alternatively, patients with long lasting CS could have forgotten specific scents and need to relearn the identification of these scents again. Then, odor training, which has been reported to improve odor discrimination and ID [27], could be a treatment option.

Furthermore, changes in the nasal epithelium by hypercortisolism could contribute to the reduced sense of smell, as it is known that glucocorticoids reduce for example inflammation in the nasal epithelium [35]. A nasal biopsy at the time of transsphenoidal surgery would be interesting.

Concerning a potential linkage between the extent of hypercortisolism and the intensity of smell and taste impairment, we found no correlation between total smell score (TDI) resp. total taste score and parameters of cortisol excess at diagnosis (24 h urinary free cortisol, cortisol after 1 mg dexamethasone and late-night salivary cortisol).

Regarding Cushing subtypes, we found non-significant differences in smell and taste scores, although patient numbers might have been too small for significance. Patients with CD showed slightly better scores than CPA patients at 3 months of follow-up. Since all patients with CD underwent endonasal-transsphenoidal surgery, the surgical procedure appears to have no adverse effect on smell and taste [36], but a longer follow-up might elucidate this further.

### Limitations of the study

This is a monocentric explorative study with a relatively small number of patients and a short follow-up. Whether hyposmia and hypogeusia are fully reversible in the long-term has to be shown by additional studies.

In conclusion, smell and taste is impaired in patients with CS. Successful surgery is able to partly restore smell and taste in the majority of patients.

## Data Availability

Some or all datasets generated during and/or analyzed during the current study are not publicly available but are available from the corresponding author on reasonable request.
